# Supplementary material for: Functionalization of Polylactide with Multiple Tetraphenyethane Inifer Groups to Form PLA Block Copolymers with Vinyl Monomers
Source: Int J Mol Sci. 2022 Dec 20;24(1):19. doi: 10.3390/ijms24010019 (PMC9820087; doi:10.3390/ijms24010019)
Supplement: Supplementary file 1 [file ijms-24-00019-s001.zip › ijms-2087200-supplementary.pdf]

SUPPORTING INFORMATION

Functionalization of Polylactide with Multiple Tetraphenyethane Inifer Groups to Form PLA Block Copolymers with Vinyl Monomers

*Mateusz Grabowski, Bartłomiej Kost, Agnieszka Bodzioch, Melania Bednarek**

Centre of Molecular and Macromolecular Studies, Polish Academy of Sciences, Sienkiewicza 112, 90-363 Lodz, Poland

1. Determination of molecular weights of synthesized PLA diols

Molecular weights of PLA polymers were calculated by the comparison of the intensities of signals (in DMSO-d_6) corresponding to -<u>CH</u>(CH_3)-OH end groups at 4.20 ppm and corresponding to -<u>CH</u>(CH_3)- groups from PLA backbone at 5.12 and 5.20 ppm (Figure 1 in the main text).

2. Analysis of PLA-based polyurethane (PLA-PU)

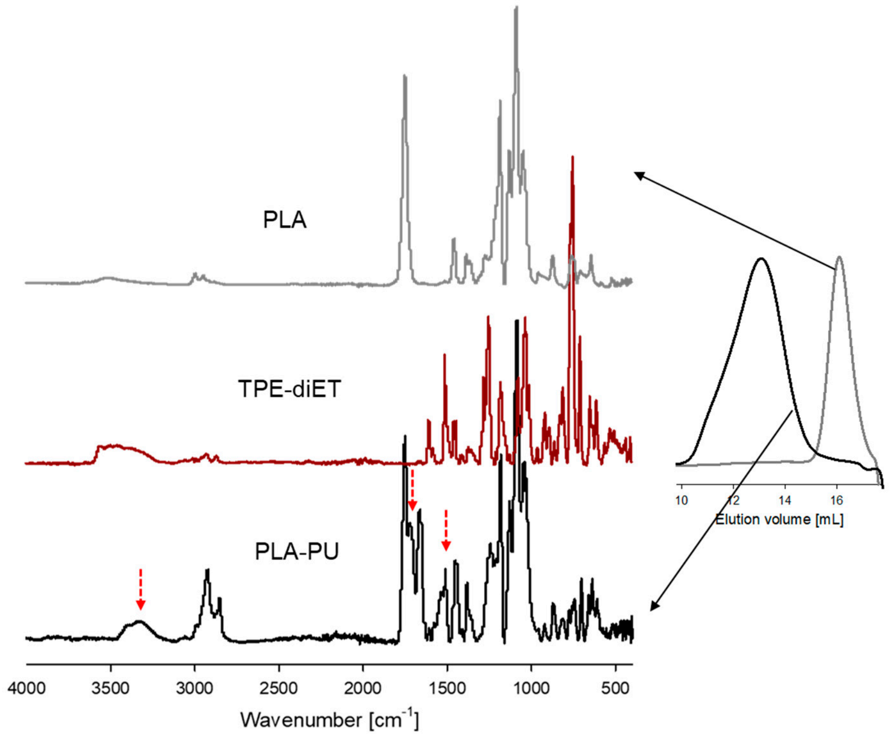

Figure S1. FTIR spectra of PLA-PU and the initial components (red arrows indicate newly formed urethane linkages) and SEC curves for starting PLA and PLA-PU (DMF as an eluent).

3. **Calculation of the relative decomposition rate of TPE groups present in PLA-PU polymer on the basis of ESR spectra**

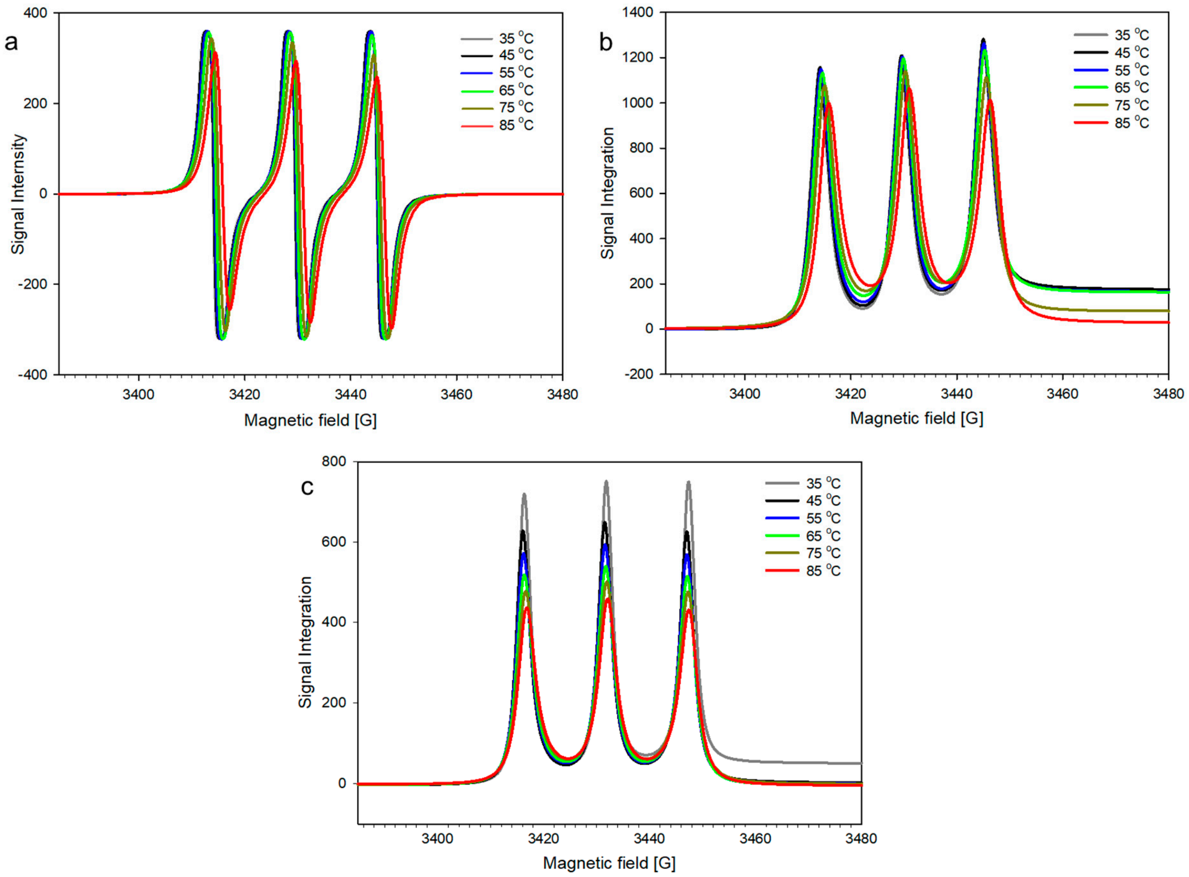

Figure S2. a) ESR spectra of TEMPO, b) normalized spectra for plots in Fig. S2a, c) normalized spectra for PLA-PU (+TEMPO) shown in Fig. 2a in the main text.

The concentration of the formed radicals was measured indirectly, by the measurement the concertation of TEMPO radicals which remained after catching …-TPE/2•. To calculate TPE decomposition ratio, the formula was applied:

$$Y = x_1(I_1 - I_2)/2x_2I_1 \quad \text{(S1a)}$$

x_1 – quantity of TEMPO (mols)

x_2 - quantity of TPE groups in PLA-PU (mols)

I_1 – integral area from the normalized absorption curve for TEMPO

I_2 – integral area from the normalized absorption curve for PLA-PU with TEMPO

Two solutions were prepared and ESR spectra for them were recorded at 35, 45, 55, 65, 75 °C:

1. 0.8 mg of TEMPO in 1 mL of DMF, i.e. 5.12 x10^{-3} mmol

2. 80 mg of PLA-PU + 0.8 mg of TEMPO in 1 mL of DMF, i.e. 46 x $x10^{-3}$ mmol + 5.12 $x10^{-3}$ mmol

After inserting the values into the equation (S1a):

$Y = 0.55(I_1 - I_2)/I_1$ (S1b)

ESR spectra presented in Fig. 2a for PU-PLA (with TEMPO) and Fig. S2a for TEMPO were transformed into normalized spectra presented in Fig. S2b and S2c using Origin Software (Integration of signals from the original ESR).

The I_1 and I_2 values were found from normalized spectra and were put into the equation 1b to find Y values at different temperatures. The dependence Y = f(T) is shown in Fig. 2b in the main text.

4. FTIR analyses of PLA/PST and PLA/PAN copolymers

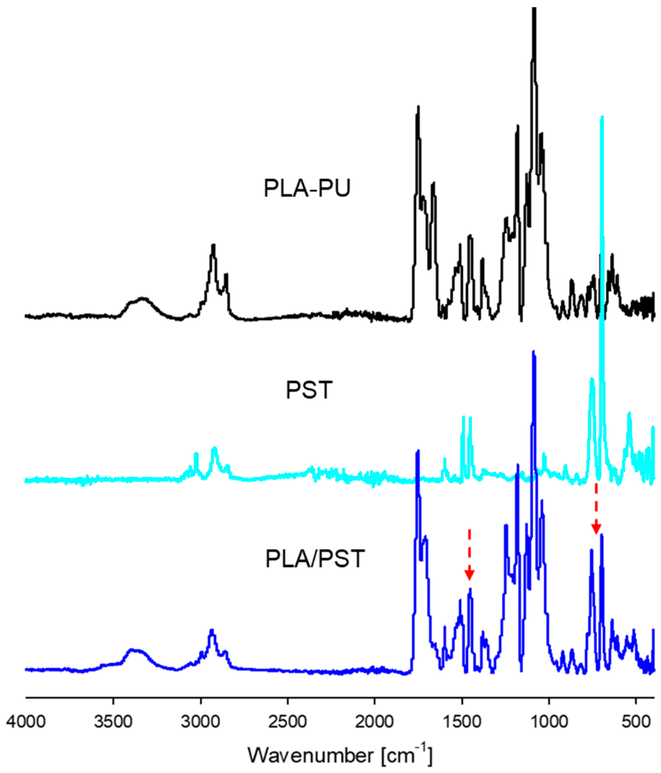

Figure S3. FTIR spectra of PLA-PU, additionally synthesized PST, and PLA/PST copolymer. Red arrows indicate the signals corresponding to aromatic ring (698 cm^{-1} and 1492 cm^{-1}) for PST [38].

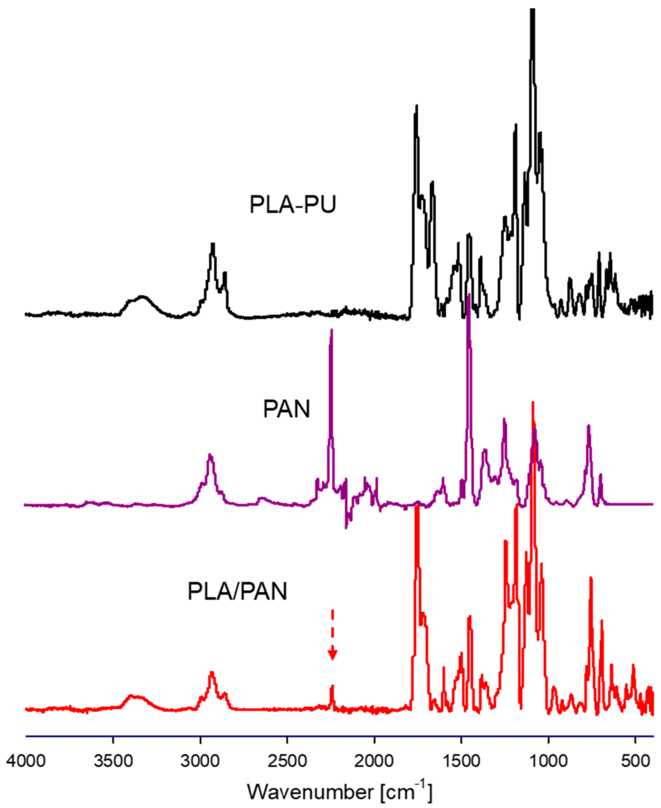

Figure S4. FTIR spectra of PLA-PU, additionally synthesized PAN, and PLA/PAN copolymer. Red arrows indicate the signal corresponding to nitrile group in PAN (2240 cm^{-1}) [39].

Homopolymers - PST and PAN for comparison in FTIR analyses were obtained via radical polymerizations of ST and AN correspondingly, initiated with AIBN.

5. The stability of PLA- based PU during heating

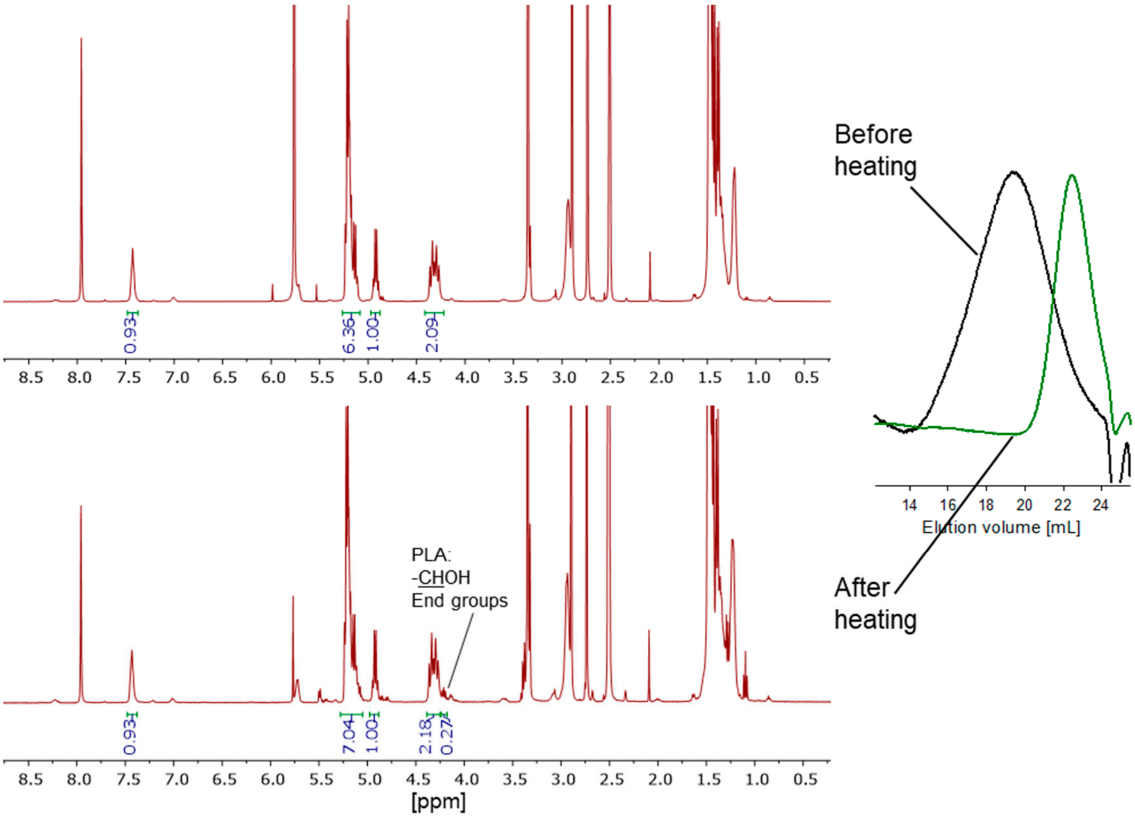

Figure S5 . ^{1}H NMR spectra of PLA-PU prepared without TPE-diET before and after heating in DMF at 85 °C for 24h and SEC curves for PU before and after heating (DMF as an eluent).

6. Diffusion coefficients of PLA-PU/PVM copolymers and corresponding homopolymers on the basis of DOSY NMR measurements

Table S1. The diffusion coefficients for groups in PLA/PVM copolymers and homopolymers corresponding to particular signals in ^{1}H NMR spectra shown in Fig. 5 and Fig. 6 in the main text.

PLA/PAN		PLA/PST	
Signal [ppm]	Diffusion coefficient x10^{7}, [cm^{2}/s]	Signal [ppm]	Diffusion coefficient x10^{7}, [cm^{2}/s]
PLA-PU		PLA-PU	
7.33-7.24<br>7.24-7.16	1.27<br>1.29	7.33-7.24<br>7.24-7.16	1.41<br>1.42

7.06-6.96<br>6.68-6.57<br>5.22-5.13<br>4.93-4.87<br>4.22-416<br>4.07-3.98<br>1.56-1.34	1.27<br>1.28<br>1.26<br>1.26<br>1.27<br>1.26<br>1.26	7.06-6.96<br>6.68-6.57<br>5.22-5.13<br>4.93-4.87<br>4.22-4.16<br>4.07-3.98<br>2.93-2.89<br>1.56-1.34	1.49<br>1.41<br>1.46<br>1.41<br>1.43<br>1.46<br>1.45<br>1.40
PAN		PST	
3.16-3.09<br>2.14-2.06	0.78<br>0.78	7.25-6.88<br>6.88-6.31<br>1.90-1.27	7.44<br>7.47<br>7.44
PLA-PU/PAN copol.		PLA-PU/PST copol	
7.76-7.02<br>6.89<br>6.64<br>5.22-5.13<br>4.93-4.87<br>4.38-4.16<br>3.22-3.05<br>2.20-1.90<br>1.51-1.41	2.80<br>2.83<br>2.82<br>2.81<br>2.78<br>2.80<br>2.85<br>2.86<br>2.76	7.75-7.62<br>7.56-7.41<br>7.23-6.30<br>5.22-5.13<br>4.93-4.87<br>4.22-416<br>2.95-2.89<br>1.56-1.34<br>1.21-1.19	3.25<br>3.20<br>3.21<br>3.21<br>3.18<br>3.27<br>3.25<br>3.26<br>3.25
